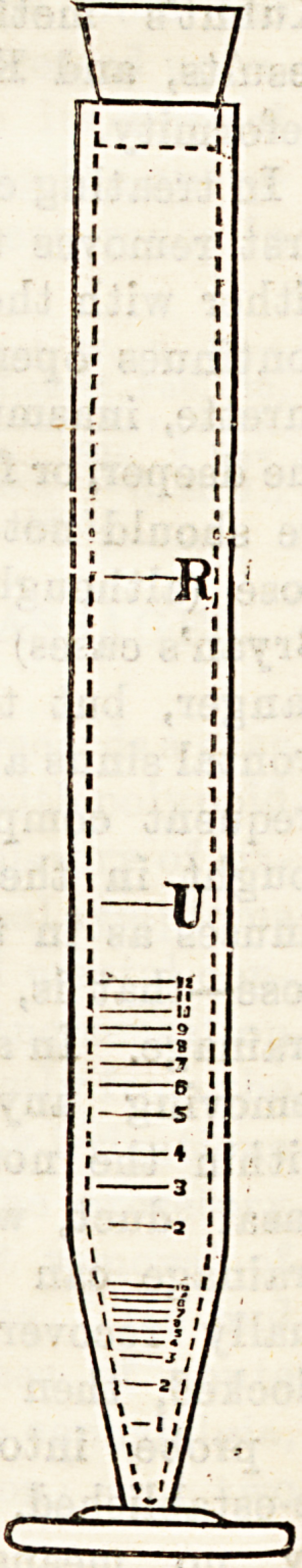# New Appliances and Things Medical

**Published:** 1896-05-09

**Authors:** 


### NEW APPLIANCES AND THINGS MEDICAL.

[We shall be glad to receive, at our Office, 428, Strand, London, W.C., from the manufacturers, specimens of all new preparations and appliances, which may be brought out from time to time.]

### KING'S OATMEAL.

(Albion Food Mills, Sycamore Street, London, E.C.)

The preparations which are manufactured by the above firm, while possessing all the qualifications of a high-class oatmeal, possess in addition the valuable property of being readily prepared. Ordinary varieties are very unpopular with those who have charge of the cooking department, since it takes something like half an hour to prepare a good porridge, and that, too, with constant attention, whereas this new cooked oatmeal is ready for use in a few minutes. All that is necessary is to mix well with milk or water to the consistency of cream, and then to throw it into a saucepan containing boiling milk or water, and boil for a minute—the porridge is then ready. King's oatmeal is sold in several qualities, fine, medium, or coarse, in tins, for 3d. or 6d.

### NEW DIETETIC PREPARATION.

(Van Abbott and Sons, 6, Duke Street Mansions, Grosvenor Square, W.)

Some new digestive biscuits for children, and those of weak digestive powers, are being prepared by the above firm. We have been forwarded a sample tin. They are reported to be free from all added chemicals, to be made of mixed flours, containing a large proportion of the whole meal in a finely divided condition, thus eliminating any irritating properties, while retaining those gently laxative effects for which whole meal preparations are usually prescribed. The biscuits are decidedly palatable, and appear to us to possess all the nutritive and laxative properties which are claimed for them by the manufacturers.

### HAYWARD'S ALBUMINOMETER.

(R. Sumner and Co., 50a, Lord's-street, Liverpool.)

This is a modified Esbach's albuminometer, and consists of a graduated glass into which the urine is poured up to the mark "U," the reagent, a solution of picric and citric acids, being added until it reaches the mark "R." After inserting an indiarubber cork provided for the purpose, the tube should be inverted two or three times so as to ensure the proper mixing of the urine and the reagent. After standing for twenty-four hours the precipitated albumin falls to the bottom and can be read off by the graduated scale. The latter is marked in grammes per litre: one gramme per litre equals ·1 per cent., so that if the total quantity of urine passed in the 24 hours is measured the total albumin can be at once calculated. The advantages of ths method are: (1) its simplicity; (2) its accuracy; albumin can be detected and measured when present in very small quantity (·01 per cent.) The price of the albuminometer is 3s., and Esbach's solution 1s. 6d. per lb.

## Figures and Tables

**Figure f1:**